# Poor Lower Extremity Function Was Associated with Pre-Diabetes and Diabetes in Older Chinese People

**DOI:** 10.1371/journal.pone.0115883

**Published:** 2014-12-22

**Authors:** Wen Zhang, Suxing Shen, Wei Wang, Chunling Zhou, Limin Xu, Jiahe Qiu, Jiaqi Wang, Xiangxue Meng, Yixiong Liang, Kaijun Niu, Qi Guo

**Affiliations:** 1 Department of Rehabilitation and Sports Medicine, Tianjin Medical University, Tianjin, China; 2 Department of Rehabilitation, Tianjin Medical University General Hospital, Tianjin, China; 3 Community Service Center, Chadian Street, Binhai New Area, Tianjin, China; 4 Nutritional Epidemiology Institute, Tianjin Medical University, Tianjin, China; 5 School of Public Health, Tianjin Medical University, Tianjin, China; 6 Department of Rehabilitation Medicine, TEDA International Cardiovascular Hospital, Cardiovascular Clinical College of Tianjin Medical University, TEDA, Tianjin, China; University of Padova, Italy

## Abstract

**Objective:**

To determine whether a relationship exists between performance-based physical assessments and pre-diabetes/diabetes in an older Chinese population.

**Methods:**

Our study population comprised 976 subjects (mean ± SD age: 67.6±6.0 years; 44.5% men) from the Hangu area of Tianjin, China. Diabetes was defined by self-reporting of a physician's diagnosis, or a fasting plasma glucose level ≥126 mg/dL; and pre-diabetes was defined as a fasting plasma glucose level ≥100 mg/dL and <126 mg/dL.

**Results:**

When all other variables were adjusted for, men needing longer to finish a Timed Up and Go Test and a decreased usual walking speed had higher odds of pre-diabetes (*P* for trend = 0.007 and 0.008, respectively) and diabetes (*P* for trend = 0.012 and 0.014, respectively). However, women needing longer to finish the test and a decreased usual walking speed had a higher odds of diabetes (*P* for trend = 0.020 and 0.034, respectively) but not of pre-diabetes. There was no apparent association between grip strength and pre-diabetes/diabetes in both sexes.

**Conclusions:**

In this study, poor lower extremity function was associated with pre-diabetes/diabetes in older people.

## Introduction

Pre-diabetes and diabetes are a growing health issue in China [1]. The symptoms of pre-diabetes and early diabetes can be subtle, especially in older adults. Thus, timely and accurate identification and prevention of blood glucose changes in this group is important. There is a lot of information available on the link between body composition and risk of diabetes [2]; however, less is known about the impact of other physiological factors such as muscle strength and physical performance. Although physical inactivity which is assessed by questionnaire, such as the International Physical Activity (IPAQ), is the fourth leading cause of death worldwide [3] which causes 7% of type 2 diabetes and 9% of premature mortality [4], whether actual physical capacity (muscle strength and physical performance) contacts to glucose levels in older adults is still unknown.

Many studies report that low muscle strength and physical dysfunction are related to pre-diabetes/diabetes in older adults. Most of them are concentrated on the effect of diabetes on worse physical capability which is assessed by walking speed [5]–[7] and grip strength [8], and that these changes are associated with loss of muscle mass and lower extremity strength [7]. Although little is known of the effects of pre-diabetes in this group, as adults with glucose intolerance and recent-onset diabetes already display microvascular and neuropathic complications [9], [10], so we have reason to believe that older adults with pre-diabetes will show lower physical performance than non-diabetic adults. In addition, the Timed Up and Go Test (TUGT) is one of the most frequently used tests of balance and gait, and is often used to assess fall risk in older adults [11], yet few studies have used the TUGT to assess the relationship between mobility and pre-diabetes/diabetes in older adults. A small-scale, cross-sectional, population survey in the Netherlands showed that a diabetic polyneuropathy group took significantly longer (by 29%) than a healthy group to complete the TUGT [12]. Thus, we have reason to believe that the TUGT is a reliable indicator of physical dysfunction in older adults with pre-diabetes/diabetes.

The objective of this study is to determine whether a relationship exists between performance-based physical assessments and pre-diabetes/diabetes in community-dwelling older Chinese people. From a public health perspective, a better understanding of physical capability is important in order to implement lifestyle interventions in real-world settings.

## Methods

### Participants

Our study population comprised residents of the Hangu area of Tianjin, China, aged ≥60 y, who joined the national free physical examination program. All subjects were invited to participate in a comprehensive geriatric assessment, with the exception of those with a disability that affected the basic activities of daily living, and thus could not carry out performance-based assessments. Of those invited, 1049 agreed to participate in the survey and gave informed written consent for data collection. The final study population comprised 976 subjects (mean ± SD age: 67.6±6.0 y; 44.5% men) after those with data deficiencies (n = 73) were excluded. This study was approved by the Ethics Committee at Tianjin Medical University, China.

### Performance-based assessment and collection of biomarker data

Performance-based assessment consisted of several physical tests. Grip strength (kg) was used as a measure of muscle strength and was quantified using a handheld dynamometer (GRIP-D; Takei Ltd, Niigata, Japan). Participants were asked to exert their maximum effort twice using their dominant hand and the average grip strength was recorded [13]. Gait function was assessed with the 4-m walk tests and the TUGT. To measure walking speed, two photocells connected to a recording chronometer were placed at the beginning and the end of a 4-meter course at the site clinic. Participants were instructed to stand with both feet touching the starting line and to begin walking at their usual pace after a verbal command was given. The time between activation of the first and the second photocell was measured and the average speed of two walks was recorded [14]. The TUGT involved rising from a chair, walking for 3 meter, turning around, walking back to the chair, and sitting down. The time taken at the participant's usual pace was measured in seconds once [15].

Blood samples were collected from all participants after an overnight fast of at least 10 h. Blood was drawn from the antecubital vein, with minimal tourniquet use, while subjects were seated. After collection samples were centrifuged for 15 min at 3000 rpm. Fasting plasma glucose (FPG), creatinine (CRE), blood urea nitrogen (BUN), total cholesterol (TC) and triglycerides (TG) were measured using the Roche Modular P (Roche Diagnostic Company, Swiss). Creatinine clearance (CCr) was calculated as [140-age (y)]*W (kg)/72*CRE (mg/ml) (15% less in females) [16].

### Assessment of diabetes

Diabetes was defined by self-reporting of a physician diagnosis, or the use of hypoglycemic medication. In undiagnosed participants, diabetes was defined as a FPG level ≥126 mg/dL and pre-diabetes as a FPG level ≥100 mg/dL but <126 mg/dL, based on the American Diabetes Association 2013 criteria [17].

### Assessment of other variables

Height and weight were recorded using a standard protocol. Body mass index was calculated as weight in kilograms divided by height in meters squared. Sociodemographic variables, including sex, age, educational level, and job were also assessed. Educational level was defined as age at completion of schooling and divided into 4 categories: illiteracy, 1–6 y, 7–12 y and ≤13 y. A fall, defined as “an unexpected event in which the person comes to rest on the ground, floor, or a lower level,” in the past year, was elicited [18]. Participants who reported multiple (>1) falls or at least one fall with injury were categorized as “fallers” [19]. A history of physical illness was evaluated on the basis of participants' response (yes or no) to questions about hypertension, hyperlipidemia, stroke, coronary heart disease (CHD), and kidney disease, including physician diagnosis, taking corresponding medication or other treatment now or in the past. Information on smoking (never, former smoker, and current smoker) and drinking (never, former drinker, everyday drinker, and occasional drinker) was obtained from a questionnaire survey. Physical activity was assessed with the short form of the International Physical Activity Questionnaire (IPAQ), in the Chinese language [20]. Responses were converted to Metabolic Equivalent Task minutes per week (MET-min/wk) [21] according to the IPAQ scoring protocol: total minutes over the previous seven days spent on vigorous activity, moderate-intensity activity, and walking were multiplied by 8.0, 4.0, and 3.3, respectively, to create MET scores for each activity level. MET scores across the three sub-categories were summed to indicate overall physical activity [21].

### Statistical analysis

Data are presented as means (with 95% confidence intervals [CIs]) or as percentages. Differences between variables were examined by ANOVA with Bonferroni correction (continuous variables) or by the chi square test (categorical variables). Logistic regression analysis was used to determine odds ratios (ORs) and 95% CIs, and to assess whether participants with pre-diabetes or diabetes independently associated with TUGT and/or walking speed when compared to those with normoglycemia. Linear regression was used for calculating p for trend in the logistic binary models. Participants were divided into 4 groups by quadrisection based on the results of grip strength measurement, TUGT, and their usual walking speed. Covariates were added sequentially to the logistic model to evaluate association at different levels of adjustment. Crude was unadjusted. Model 1 was adjusted for age, BMI, hypertension, hyperlipidemia, stroke, CHD, kidney disease, whether the participant was a farmer, educational level, history of smoking and drinking, history of falls, IPAQ score, CRE, BUN, TC, and TG. Model 2 was adjusted for model 1 variables as well as all other performance-based assessments. The interactions between pre-diabetes/diabetes and all confounders for each performance-based assessment were tested through the addition of the cross-product terms to the regression model. Differences were defined as significant when P<0.05. All statistical analyses were performed with the SPSS V19.0 software package (SPSS Inc, China).

## Results

### Subject characteristics

Of the 976 subjects in this study, 29.2% (285/976) [31.3% (136/434) of men and 27.5% (149/542) of women] were classified as having pre-diabetes and 14.4% (141/976) [11.1% (48/434) of men and 17.2% (93/542) of women] as having diabetes; 48 subjects (men 25, women 23) were newly diagnosed with diabetes based on FPG levels ≥126 mg/dL.

Data for each category is presented in **Table 1**. Participants with diabetes were more likely than normoglycemic participants to have a lower level of education, history of smoke, with higher TC, hypertension, and hyperlipidemia and lower CCr among men; and, a lower level of education, with higher BMI, hypertension, hyperlipidemia, and CHD among women. We also found that older adults with diabetes were more likely than normoglycemic participants to have a history of falls in the past year, as assessed by self-reporting (men: *P*<0.05; women: *P*<0.05). In addition, men with pre-diabetes were more likely to be ex-smokers than those in the other two groups (*P* = 0.017).

**Table 1:** Subject characteristics according to gander and categories of pathoglycemia.

	Male				Female			
	Normoglycemia (n = 250)	Prediabetes (n = 136)	Diabetes (n = 48)	P value	Normoglycemia (n = 300)	Prediabetes (n = 149)	Diabetes (n = 93)	P value
**Age, y**	68.1 (67.3–68.9)	69.6 (68.3–70.8)*	69.2 (67.1–71.3)	0.099	66.4 (65.8–67.0)	66.5 (65.6–67.3)	67.8 (66.7–68.9)*	0.086
**BMI, kg/m^2^**	24.8 (24.4–25.3)	25.2 (24.7–25.7)	24.9 (23.1–26.6)	0.662	24.9 (24.5–25.4)	25.9 (25.2–26.6)*	26.4 (25.6–27.1)*	0.002
**Farmer, %(n)**	78.8 (197)	84.6 (115)	72.9 (35)	0.175	89.0 (267)	91.9 (137)	92.5 (86)	0.461
**Educational level, %(n)**								
** Illiteracy**	18.0 (45)	21.3 (29)	14.6 (7)	0.540	25.0 (75)	32.3 (30)*	38.3 (57)*	0.013
**1**–**6 y**	55.6 (139)	59.6 (81)	62.5 (30)	0.578	62.7 (188)	51.7 (77)	53.8 (50)	0.055
**7**–**12 y**	26.0 (65)	18.4 (25)	20.8 (10)	0.220	12.0 (36)	9.4 (14)	14.0 (13)	0.532
**≥13 y**	3.6 (9)	2.2 (3)	10.4 (5)*^†^	0.039	3.3 (10)	2.7 (4)	0 (0)	0.208
**Smoke status, %(n)**								
** Current smoker**	39.6 (99)	33.1 (45)	33.3 (16)	0.460	33.7 (101)	28.2 (42)	21.5 (20)	0.069
** Ex-smoker**	30.0 (75)	30.1 (41)	39.6 (19)*^†^	0.017	12.0 (36)	9.4 (14)	14.0 (13)	0.532
** Nonsmoker**	30.4 (76)	36.8 (50)	27.1 (13)	0.325	54.0 (163)	62.4 (93)	64.5 (60)	0.108
**Drinking status, %(n)**								
** Drink everyday**	16.8 (42)	14.0 (19)	18.8 (9)	0.672	13.7 (41)	14.1 (21)	11.8 (11)	0.872
** Drink occasionally**	14.4 (36)	16.2 (22)	16.7 (8)	0.859	16.3 (49)	14.1 (21)	10.8 (10)	0.401
** x-drinker**	8.4 (21)	13.2 (18)	10.4 (5)	0.322	10.7 (32)	10.7 (16)	10.8 (10)	0.978
** Nondrinker**	60.4 (151)	56.6 (77)	54.2 (26)	0.625	59.3 (178)	61.1 (91)	66.7 (62)	0.448
**Fallers, %(n)**	3.2 (8)	4.4 (6)	4.2 (2)	0.591	6.0 (18)	5.4 (8)	8.6 (8)	0.488
**IPAQ, Met/week**	4035.3 (3524.4–4546.0)	3783.4 (3153.2–4413.7)	3730.4 (2588.0–4872.8)	0.786	3409.0 (3002.8–3815.2)	3273.0 (2725.5–3820.5)	2734.9 (2005.4–3464.3)	0.272
**Blood index**								
** CCr, min/L**	90.1 (80.4–95.8)	77.2 (65.2–79.2)*	70.4 (64.1–73.3)*	0.045	90.0 (79.7–93.3)	88.0 (78.7–90.3)	83.5 (72.3–86.7)	0.063
** BUN, mmol/L**	5.6 (5.4–5.7)	5.7 (5.5–6.0)	5.4 (4.9–5.9)	0.401	5.4 (5.2–5.5)	5.3 (5.1–5.6)	5.4 (5.1–5.6)	0.995
** TC, mmol/L**	4.6 (4.4–4.7)	4.8 (4.6–5.0)	8.4 (3.3–13.6)*^†^	<0.001	5.2 (5.1–5.4)	5.3 (4.8–5.7)	5.9 (4.2–7.5)	0.381
** TG, mmol/L**	1.4 (1.3–1.5)	2.5 (0.4–4.6)	4.1 (1.8–6.4)*	0.053	2.2 (1.1–3.4)	3.0 (0.9–5.1)	5.7 (1.7–13.2)	0.265
**Hypertension, %(n)**	30.4 (76)	45.6 (62)*	45.8 (22)*	0.005	42.0 (126)	52.3 (78)*	68.8 (64)*	<0.001
**Hyperlipidemia, %(n)**	5.6 (14)	4.4 (6)	14.6 (7)*^†^	0.035	7.7 (23)	6.7 (10)	20.4 (19)*^†^	<0.001
**Stroke, %(n)**	8.8 (22)	8.8 (12)	6.3 (3)	0.836	6.3 (19)	4.7 (7)	9.7 (9)	0.306
**CHD, %(n)**	14.4 (36)	16.9 (23)	22.9 (11)	0.325	26.0 (78)	30.2 (45)*	48.4 (45)*	<0.001
**Kidney disease, %(n)**	2.8 (7)	2.9 (4)	4.2 (2)	0.878	3.3 (10)	2.7 (4)	8.6 (8)	0.081

1. Pre-diabetes is defined by having fasting plasma glucose (FPG) levels ≥100 mg/dl (5.6 mmol/L) but <126 mg/dl (7.0 mmol/L); diabetes is defined by self-reported or FPG ≥126 mg/dl (7.0 mmol/l)

2. BMI, body mass index; CCr, creatinine clearance; BUN, blood urea nitrogen; TC, total cholesterol; TG, triglyceride; CHD, coronary heart disease; IPAQ, international physical activity questionnaire.

3. Obtained by using ANOVA for continuous variables and chi-square for variables of proportion.

4. Mean; 95% CI in parentheses (all such values)

5. * normoglycemia compare with prediabetes & diabetes; ^†^prediabetes compare with diabetes (P<0.05)

### Physical performance and pre-diabetes/diabetes

**Table 2** shows the ORs determined from logistic regression analyses of the quartiles of grip, TUGT, or usual walking speed, for each group. Firstly, we observed significant interaction between the outcomes of grip or TUGT or usual walking speed and sex either for pre-diabetes (grip P for interaction = 0.009; TUGT P for interaction = 0.019; usual walking speed P for interaction = 0.024), but not for pre-diabetes and diabetes or for diabetes (grip P for interaction: 0.299 for pre-diabetes and diabetes, 0.735 for diabetes; TUGT P for interaction: 0.812 for pre-diabetes and diabetes, 0.779 for diabetes; usual walking speed P for interaction: 0.508 for pre-diabetes and diabetes, 0.885 for diabetes). Furthermore, physical performance items are different between men and women because of different physiological features [22], [23], so we categorize participants according to sex. In the crude analysis, men that took longer to finish the TUGT, and had a lower than usual walking speed, had a higher chance of pre-diabetes (*P* for trend = 0.006 and 0.034, respectively). The same was found in the model 1 (*P* for trend = 0.001 and 0.008, respectively) and model 2 (*P* for trend = 0.007 and 0.004, respectively). In women from this category, there were no statistically significant differences between pre-diabetes and normoglycemia. In contrast, there was a consistently higher chance of diabetes among both men and women in this category, based on both the crude model (men: *P* for trend = 0.002 and 0.029, respectively; women: *P* for trend = 0.018 and 0.027, respectively), model 1 (men: *P* for trend = 0.025 and 0.036, respectively; women: *P* for trend = 0.039 and 0.031, respectively) and model 2 (men: *P* for trend = 0.012 and 0.014, respectively; women: *P* for trend = 0.020 and 0.034, respectively). Finally, we also found that men who took longer to finish the TUGT, and had a lower than usual walking speed, had a higher chance of both pre-diabetes and diabetes based on the crude model (*P* for trend = 0.014 and 0.006, respectively), model 1 (*P* for trend = 0.013 and 0.003, respectively) and model 2 (*P* for trend = 0.014 and 0.017, respectively). There was no apparent association between grip and pre-diabetes/diabetes in men and women.

**Table 2:** Logistic regression analyses of grip, TUGT and usual walking speed quartiles association with pathoglycemia (prediabetes and diabetes).

	Male					Female				
	Q1	Q2	Q3	Q4	*P* for trend^2^	Q1	Q2	Q3	Q4	*P* for trend^2^
**Grip**										
**Prediabetes**										
n (%)	39 (40.2)	32 (33.3)	30 (30.6)	35 (37.2)		29 (25.9)	38 (32.2)	42 (38.5)	40 (36.4)	
Crude	1.00	0.74 (0.41–1.34)	0.66 (0.36–1.19)	0.88 (0.49–1.58)	0.617	1.00	1.36 (0.77–2.41)	1.79 (1.01–3.18)	1.64 (0.92–2.90)	0.146
Model 1	1.00	0.78 (0.34–1.81)	0.82 (0.35–1.93)	1.28 (0.50–3.27)	0.507	1.00	1.05 (0.49–2.27)	2.26 (1.04–4.90)	1.78 (0.77–4.08)	0.278
Model 2	1.00	0.79 (0.34–1.87)	0.87 (0.37–2.06)	1.32 (0.51–3.37)	0.431	1.00	1.10 (0.50–2.40)	2.44 (1.11–5.39)	1.89 (0.81–4.44)	0.275
**Diabetes**										
n (%)	19 (25.7)	11 (14.3)	8 (10.8)	10 (13.9)		31 (31.3)	24 (24.5)	19 (19.2)	19 (19.6)	
Crude	1.00	0.49 (0.18–1.35)	0.43 (0.15–1.24)	0.40 (0.13–1.23)	0.124	1.00	0.58 (0.27–1.22)	0.60 (0.28–1.26)	0.50 (0.23–1.11)	0.137
Model 1	1.00	0.43 (0.10–1.91)	0.14 (0.02–0.85)	0.24 (0.40–1.49)	0.120	1.00	0.62 (0.25–1.56)	1.02 (0.38–2.69)	0.64 (0.22–1.89)	0.555
Model 2	1.00	0.45 (0.10–2.07)	0.14 (0.02–0.90)	0.27 (0.04–1.73)	0.132	1.00	0.64 (0.25–1.63)	1.14 (0.42–3.06)	0.73 (0.24–2.17)	0.778
**Prediabetes+diabetes**										
n (%)	53 (48.6)	44 (40.7)	41 (37.6)	46 (42.6)		60 (43.2)	57 (42.5)	66 (49.3)	59 (43.7)	
Crude	1.00	0.67 (0.36–1.27)	0.59 (0.31–1.12)	0.71 (0.37–1.35)	0.296	1.00	0.90 (0.52–1.57)	1.61 (0.92–2.81)	1.01 (0.57–1.79)	0.760
Model 1	1.00	0.88 (0.40–1.91)	0.79 (0.36–1.74)	1.00 (0.42–2.40)	0.876	1.00	0.84 (0.44–1.58)	1.89 (0.97–3.68)	1.17 (0.58–2.38)	0.618
Model 2	1.00	0.91 (0.42–2.00)	0.81 (0.37–1.80)	1.06 (0.44–2.54)	0.912	1.00	0.89 (0.47–1.70)	2.07 (1.05–4.08)	1.27 (0.62–2.61)	0.569
**TUGT**										
**Prediabetes**										
n (%)	25 (25.8)	30 (31.6)	31 (36.5)	50 (45.9)		37 (31.1)	35 (25.2)	43 (43.9)	34 (36.6)	
Crude	1.00	1.33 (0.71–2.49)	1.65 (0.88–3.12)	2.44 (1.35–4.41)	0.006	1.00	0.75 (0.43–1.29)	1.73 (0.99–3.02)	1.28 (0.72–2.27)	0.527
Model 1	1.00	1.29 (0.65–2.54)	1.54 (0.76–3.12)	2.04 (1.00–4.18)	0.001	1.00	0.68 (0.37–1.25)	1.59 (0.85–2.99)	1.18 (0.58–2.40)	0.590
Model 2	1.00	1.32 (0.65–2.67)	1.60 (0.74–3.46)	2.36 (0.96–5.80)	0.007	1.00	0.76 (0.40–1.45)	2.01 (0.96–4.21)	1.74 (0.69–4.40)	0.301
**Diabetes**										
n (%)	10 (13.7)	9 (12.0)	13 (17.6)	16 (21.1)		12 (12.1)	17 (17.3)	32 (31.7)	32 (33.7)	
Crude	1.00	1.16 (0.33–2.25)	1.34 (0.55–3.29)	1.68 (0.71–3.99)	0.002	1.00	1.52 (0.69–3.38)	3.36 (1.61–7.01)	3.68 (1.76–7.71)	0.018
Model 1	1.00	1.15 (0.19–2.22)	1.73 (0.54–5.52)	2.14 (0.66–6.94)	0.025	1.00	1.41 (0.54–3.69)	3.26 (1.34–7.94)	3.32 (1.26–8.75)	0.039
Model 2	1.00	1.13 (0.18–2.22)	1.31 (0.42–5.03)	1.46 (0.33–6.06)	0.012	1.00	1.41 (0.53–3.75)	3.70 (1.43–9.61)	4.15 (1.28–13.43)	0.020
**Prediabetes+diabetes**										
n (%)	36 (33.0)	40 (36.7)	49 (45.4)	59 (54.6)		51 (37.2)	45 (33.6)	76 (55.1)	70 (52.6)	
Crude	1.00	1.18 (0.67–2.05)	1.68 (0.97–2.92)	2.44 (1.41–4.23)	0.014	1.00	0.85 (0.52–1.40)	2.07 (1.28–3.35)	1.87 (1.15–3.05)	0.244
Model 1	1.00	1.19 (0.64–2.16)	1.64 (0.89–3.05)	2.39 (1.21–4.71)	0.013	1.00	0.73 (0.42–1.27)	1.88 (1.09–3.25)	1.66 (0.90–3.04)	0.306
Model 2	1.00	1.22 (0.65–2.26)	1.74 (0.90–3.35)	2.66 (1.19–5.96)	0.014	1.00	0.82 (0.46–1.46)	2.28 (1.23–4.22)	2.31 (1.07–5.01)	0.169
**Usual walking speed**										
**Prediabetes**										
n (%)	39 (44.8)	29 (34.9)	34 (34.7)	34 (28.8)		38 (36.9)	47 (34.1)	33 (27.7)	31 (34.8)	
Crude	1.00	0.75 (0.36–1.58)	0.62 (0.31–1.23)	0.54 (0.27–1.05)	0.034	1.00	0.95 (0.50–1.82)	0.62 (0.31–1.24)	0.98 (0.48–2.02)	0.803
Model 2	1.00	0.83 (0.46–1.80)	0.77 (0.45–1.58)	0.63 (0.38–1.26)	0.008	1.00	0.93 (0.43–2.00)	0.56 (0.25–1.27)	1.01 (0.42–2.46)	0.880
Model 3	1.00	0.82 (0.56–2.17)	0.70 (0.52–1.79)	0.57 (0.47–1.64)	0.004	1.00	1.04 (0.45–2.40)	0.72 (0.27–1.87)	1.27 (0.42–3.91)	0.639
**Diabetes**										
n (%)	16 (21.1)	13 (17.3)	10 (13.3)	9 (12.5)		36 (35.6)	20 (19.6)	17 (17.7)	20 (21.3)	
Crude	1.00	0.73 (0.26–2.11)	0.70 (0.25–1.94)	0.49 (0.15–1.55)	0.029	1.00	0.45 (0.16–0.76)	0.38 (0.19–0.80)	0.31 (0.14–0.72)	0.027
Model 1	1.00	0.82 (0.25–2.04)	0.74 (0.17–2.30)	0.34 (0.05–1.45)	0.036	1.00	0.47 (0.18–1.23)	0.39 (0.15–1.04)	0.36 (0.17–1.39)	0.031
Model 2	1.00	0.83 (0.39–2.82)	0.70 (0.27–2.69)	0.53 (0.08–1.58)	0.014	1.00	0.53 (0.19–1.47)	0.48 (0.15–1.48)	0.43 (0.18–2.35)	0.034
**Prediabetes+diabetes**										
n (%)	38 (56.7)	70 (44.0)	39 (38.6)	37 (34.6)		74 (53.2)	61 (44.5)	51 (37.5)	56 (43.1)	
Crude	1.00	0.80 (0.41–1.54)	0.61 (0.30–1.26)	0.50 (0.24–1.03)	0.006	1.00	0.65 (0.37–1.16)	0.52 (0.30–0.90)	0.56 (0.31–1.02)	0.177
Model 1	1.00	0.84 (0.63–2.29)	0.66 (0.50–2.20)	0.52 (0.36–1.35)	0.003	1.00	0.68 (0.35–1.33)	0.49 (0.25–0.97)	0.64 (0.31–1.33)	0.281
Model 2	1.00	0.87 (0.85–2.53)	0.74 (0.74–2.75)	0.51 (0.56–1.78)	0.017	1.00	0.75 (0.37–1.53)	0.59 (0.27–1.29)	0.78 (0.32–1.92)	0.427

1. TUGT, time up and go test

2. ORs were determined from logistic regression analyses for the quartiles of grip or TUGT or usual walking speed, comparing participants with pathoglycemia (prediabetes and diabetes) to those with normoglycemia.

3. Crude: no adjustment; Model 1: adjusted for age; body mass index (BMI); hypertension; hyperlipidemia; stroke; coronary heart disease (CHD); kidney disease; having 2 or more chronic diseases; whether famer or not; educational level; history of smoking and drinking habits; history of falls; physical activity (IPAQ); creatinine (CRE); blood urea nitrogen (BUN); total cholesterol (TC); triglyceride (TG); Model 2: adjusted for Model 1 variables in addition to the other performance-based assessments.

4. Adjusted odds ratio; 95% CI in parentheses

## Discussion

Our findings suggest a difference in the risk of falling, and in usual walking speed, between normoglycemia and diabetes in both men and women. It also seems likely that men who have a high risk of falling and a lower usual walking speed were significantly associated with pre-diabetes; however, this relationship was not seen in women. The main strength of the present study is our identification of a difference in physical performance, as assessed by objective measures of physical capability, between not only in patients with diabetes, but also those with pre-diabetes and normoglycemia. Our results can supplement research on the relationship between physical performance and glucose level for both sexes of community-dwelling older adults. Additionally, we have validated three different performance-based assessments that can determine actual physical capacity and predict subsequent physical limitations in this demographic.

As with prior studies that identified individuals with physical dysfunction based on the 4-m walking test [7], we found an association between a lower than usual walking speed and diabetes in older men and women. Several factors may contribute to this decline, including the loss of lower extremity strength and reduced muscle quality [7] by reason of diabetic neuropathy [24]; peripheral arterial disease [25]; increased muscle fat infiltration [26]; and the level of inflammatory cytokines such as THF-a and IL-6 [27], [28]. However, there is little evidence describing the difference in physical performance between pre-diabetes and normoglycemia among community-living older adults. Our finding suggests that physical dysfunction based on the 4-m walking test was associated with pre-diabetes among older men, it is consistent with a previous report showing that physical dysfunction, based on self-reporting, is prevalent among middle-aged and older Americans with pre-diabetes [29]. However, in women, the relationship between physical dysfunction and pre-diabetes was less obvious.

Our study also indicates that, when compared to the normoglycemia and diabetes groups, there is less of an association between pre-diabetes and lower usual walking speeds, among women than men. This is consistent with a study on older adults in Britain that suggested a graded association between increasing glucose levels, weaker muscle strength, and physical dysfunction, however, this was seen only in men, not in women [30]. The pathogenesis of these results is still unclear. Leigh et al. have reported [31] that the transition from simple obesity to pre-diabetes is likely accompanied by a more dramatic reduction in skeletal muscle glucose metabolism in men than in women. And they believed this is related to a lower intramuscular triglyceride oxidation and turnover rate in men, with subsequent accumulation of intramuscular triglycerides [31]. This metabolic difference between sexes may be the reason for lower muscle quality and physical performance only in men with pre-diabetes. This study also indicated that in the pre-diabetic state, men have lower insulin sensitivity than women, possibly due to lower oxidative capacity and/or synthesis of the diacylglycerol (DAG) pool [31]. Thus, altered intramuscular lipid metabolism likely occurs later in diabetes development in women than in men. Future studies are recommended to examine the differences among the sexes in the mechanisms underlying the differential effects of glycemic abnormalities on physical function.

The TUGT has been recommended as a screening tool for older adults who are at risk of falling [32]. Although a relationship between the TUGT and diabetes is not well established, a relationship between diabetes and falls in older adults has been proven [33], [34]. There are various phenotypes linked with aging, such as cognitive impairment and muscle weakness, resulting in lower TUGT scores and higher fall risk, especially in people with pre-diabetes/diabetes [35]–[37]. In this study, older adults with problems with balance and gait, as assessed by the TUGT was associated with diabetes. Moreover, in agreement with our findings on walking speed in pre-diabetes, there was a smaller difference in TUGT results between pre-diabetes and normoglycemia for women than men, and this may also be connected to sex differences in the progression of diabetes. In addition, a previous study reports that lower walking speed may predict falls in older adults, because walking speed reflects overall health and functional status [34]. However, after adjusting for walking speed, as assessed by the 4-m walking test, we still see a link between TUGT time and pre-diabetes/diabetes. So we have reason to believe that the results of the TUGT is independently associated with pre-diabetes/diabetes. However, older adults who show greater postural sways are more likely had pre-diabetes/diabetes, on which further study is needed as information concerning the underlying mechanisms is contradictory [38].

Older adults who have lower than normal lower extremity strength and function are likely to have diabetes, but our study found no difference in grip strength between older men and women with pre-diabetes/diabetes and those without, which corroborates previous reports [5]. This could be because the function of lower extremities are predominantly involved in diabetic neuropathy, presumably due to a length-dependent degeneration of nerve fibers [39], [40], and the presence and severity of peripheral neuropathy is related to the muscle strength in diabetic patients [25]. Recognizing that older adults who had below-normal lower extremity function rather than upper extremity function are more likely to have pre-diabetes/diabetes, may provide a measure that is more predictive of progressive and catastrophic disability and mobility problems [41]. Further research should identify the role of reduced skeletal muscle strength and quality in subjects with risk of pre-diabetes/diabetes.

Our study has several limitations. First, it was a cross-sectional study, and thus we could not conclude whether pre-diabetes/diabetes led to an increase in the occurrence of physical dysfunction, or vice versa. Therefore, further study should be undertaken to elucidate this relationship. Second, as the assessments were performed in a public facility, participants were more likely to be active and healthy. Therefore, our results might not be fully representative of the general elderly population. Prospective studies should be multifactorial, include more participants, and carry out more comprehensive assessments to further enhance our understanding of the relationship between pre-diabetes/diabetes and physical dysfunction. Third, we didn't involved the duration of disease or the presence or not of complications as criterion for defining or not these conditions. Although some articles had reported the relationship between the severity and the duration of diabetes, the degree of glycaemic control and the risk factors in relation to the duration of T2DM followed different patterns. Franch-Nadal J, et al had reported that diabetes duration was associated with a poorer glycaemic control but in general had a limited role in blood pressure control or lipid profile based on the data of 3130 patients [42]. Therefore, further study should be undertaken to elucidate whether metabolic control and cardiovascular risk factors in type 2 diabetes mellitus patients according to diabetes duration. Forth, there are some physical performance measures which we didn't considered, such as Walking Impairment Questionnaire (WIQ). WIQ has been demonstrated that it can be used for assessing the correlation between lower extremity performance in type 2 diabetic patients [43]. In the future, we will add relevant data in our subsequent study.

In summary, lower extremity function is likely associated with pre-diabetes/diabetes in older people. These results also highlight the importance of measuring functional outcomes, especially TUGT and usual walking speed test, in clinical studies of pre-diabetes/diabetes, to enable development of therapies to prevent pre-diabetes/diabetes in older adults with physical dysfunction.
